# Evaluation of the EMulate Therapeutics Voyager’s ultra-low radiofrequency energy in murine model of glioblastoma

**DOI:** 10.1186/s42234-024-00143-8

**Published:** 2024-04-10

**Authors:** Rajesh Mukthavaram, Pengfei Jiang, Sandra Pastorino, Natsuko Nomura, Feng Lin, Santosh Kesari

**Affiliations:** 1 Neuro-Oncology Program, Moores Cancer Center, UC San Diego, La Jolla, CA USA; 2 Department of Translational Neurosciences, Pacific Neuroscience Institute & Saint John’s Cancer Institute at Providence Saint John’s Health Center, 2200 Santa Monica Blvd., Santa Monica, CA 90404 USA; 3 Curescience Institute, 5820 Oberlin Drive Ste 202, San Diego, CA 92121 USA

**Keywords:** Ultra-low radiofrequency energy, Glioblastoma, Xenograft model, Medical device, Magnetic fields, Novel therapy

## Abstract

**Background:**

Glioblastoma (GBM) presents as an aggressive brain cancer, notorious for its recurrence and resistance to conventional treatments. This study aimed to assess the efficacy of the EMulate Therapeutics Voyager®, a non-invasive, non-thermal, non-ionizing, battery-operated, portable experimental medical device, in treating GBM. Using ultra-low radiofrequency energy (ulRFE) to modulate intracellular activity, previous preliminary results in patients have been encouraging. Now, with a focus on murine models, our investigation seeks to elucidate the device's mechanistic impacts, further optimizing its therapeutic potential and understanding its limitations.

**Methods:**

The device employs a silicone over molded coil to deliver oscillating magnetic fields, which are believed to interact with and disrupt cellular targets. These fields are derived from the magnetic fluctuations of solvated molecules. Xenograft and syngeneic murine models were chosen for the study. Mice were injected with U-87 MG or GL261 glioma cells in their flanks and were subsequently treated with one of two ulRFE cognates: A1A, inspired by paclitaxel, or A2, based on murine siRNA targeting CTLA4 + PD1. A separate group of untreated mice was maintained as controls.

**Results:**

Mice that underwent treatments with either A1A or A2 exhibited significantly reduced tumor sizes when compared to the untreated cohort.

**Conclusion:**

The EMulate Therapeutics Voyager® demonstrates promising potential in inhibiting glioma cells in vivo through its unique ulRFE technology and should be further studied in terms of biological effects in vitro and in vivo.

## Background

Glioblastoma (GBM) stands as one of the most formidable challenges in oncology. Classified as an aggressive, malignant brain tumor, GBM is known for its rapid progression and resistance to conventional treatments (Louis et al. 2016; Ostrom et al. 2017). For patients diagnosed with this condition, the prognosis remains dismal; the median survival period post-diagnosis is a scant 15 months, underscoring the urgency of identifying innovative therapeutic strategies (Sim et al. 2018; Stupp et al. 2005; Stupp et al. 2017). Traditional therapeutic approaches, although standardized, have met with limited success, amplifying the need for novel treatment modalities to ameliorate patient outcomes.

In this evolving landscape, the EMulate Therapeutics Voyager® has emerged as promising technology. An experimental medical device, the Voyager leverages the power of localized, ultra-low (0-22kHz) radio frequency energy (ulRFE) to inhibit cancers including GBM (Barkhoudarian et al. 2023; Cobbs et al. 2019; Murphy et al. 2019; Ulasov et al. 2017). At the heart of this technology are unique ulRFE signals, termed cognates. These cognates owe their genesis to a sophisticated, ultrasensitive magnetometer, designed to detect and record the subtle alterations in magnetic fields generated by solvated molecules (Cobbs et al. 2019; Murphy et al. 2019; Ulasov et al. 2017; Butters et al. 2014). Diving deeper into the spectrum of cognates, the ulRFE cognate A1A, inspired by the dynamics of paclitaxel, is hypothesized to hinder cell division and proliferation (Butters et al. 2014). On the other hand, the ulRFE cognate A2, conceptualized from murine siRNA sequences, bears the potential to suppress CTLA-4 and PD-1 expression, pivotal checkpoints in the immune response (Barber et al. 2006; Pedicord et al. 2011). A significant advantage of utilizing magnetic fields, as orchestrated by the Voyager, lies in their ability to traverse bone and a plethora of tissues without discernible attenuation, and more importantly, sans posing any health threats (Barnes and Greenebaum 2007; International Comission on Non-ionizing Radiation Protection (ICNIRP) 1998).

Using ulRFE to regulate intracellular processes, we've seen promising outcomes in our initial patient studies. As we shift our focus to murine models, our aim is to delve deeper into the device’s underlying mechanisms, refining its therapeutic potential, and pinpointing any limitations. Presently, murine glioblastoma models are predominantly divided into three categories: xenograft models using human-derived cells, genetically modified models resulting from specific genetic alterations, and syngeneic models where murine cancer cells are introduced to a host of the identical species (Kijima and Kanemura 2017). In an earlier research endeavor, the efficacy of a ulRFE cognate targeting EGFR was established, demonstrating promising results in an orthotopic xenograft GBM model (Ulasov et al. 2017). Building on this groundwork, our ongoing research employs both flank xenograft and syngeneic GBM models to explore the therapeutic efficacy of two unique ulRFE cognates.

## Methods

### Device overview

**Fig. 1:**
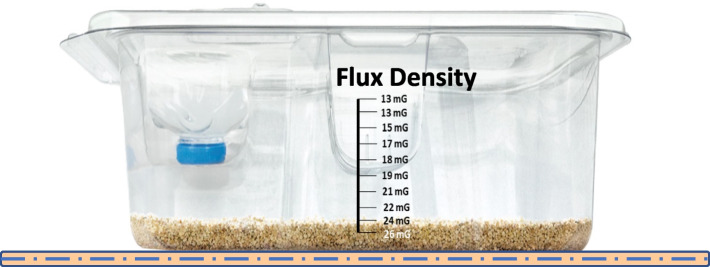
EMulate Therapeutics Voyager system integrated with mouse cages. The Voyager mouse cage coil is placed under the mouse cage and is connected to the Voyager controller. The portable controller is placed in a convenient location near the mouse cage and transmits the cognate to the mouse cage coil, which emits an oscillating magnetic field with an average field strength of ~ 25 mGauss that envelops the mouse cage

The EMulate Therapeutics Voyager system, as explored in this study was produced for EMulate Therapeutics Inc. by Omnica Corporation, based in Irvine, CA. This system comprises three main elements: the Voyager controller, the Voyager mouse cage coil, and an AC charger. A more in-depth description is provided in reference (Figueroa et al. 2022). The Voyager mouse cage coil is positioned beneath the mouse cage and connects to the Voyager controller as shown in Fig. 1. The compact controller is conveniently situated near the mouse cage and relays the cognate to the cage coil. In turn, this coil produces an oscillating magnetic with an average intensity of about 25 mGauss that surrounds the mouse cage. Throughout the study, mice remained within this magnetic field almost uninterrupted, with breaks only for cage maintenance and essential handling.

### Cognates

In these murine experiments conducted, two specific ulRFE cognates, namely A1A and A2. A1A were utilized:

- A1A: This ulRFE cognate represents the electrostatic surface potential of paclitaxel dissolved in Cremophor® EL with 49.7% dehydrated ethanol, at a concentration of 6 mg/mL paclitaxel (NDC 0015–3475-30). Its creation involved the use of 226 mV of DC offset, resulting in a static field of approximately 100 mG. This information was archived in a 16-bit, pulse code modulated WAV file format, with a frequency bandwidth ranging from DC up to 22 kHz.
- A2: This ulRFE cognate was recorded from siRNA specific to murine cytotoxic T-lymphocyte antigen 4 (CTLA-4) and programmed death 1 (PD-1) immune checkpoint receptors. The lyophilized powder of each siRNA was obtained from Integrated DNA Technologies (San Diego, CA) and resuspended in water to 40 uM final concentration. The individual siRNA recordings were concatenated to yield the A2 cognate.

### Xenograft model

Murine studies were conducted at the University of California—San Diego (UCSD) Animal Vivarium, located in the Moores Cancer Research Center. These experiments were carried out under the oversight and approval of the local Institutional Animal Care and Use Committee (IACUC), adhering strictly to UCSD’s established protocols (protocol #140220). The mice had unrestricted access to both food and water and were monitored daily for clinical observations.

The U-87 MG cell line was generously provided by Dr. Charles Stiles from the Dana-Farber Cancer Institute. These cells were cultivated in a CO2 incubator using Eagle’s minimum essential medium. This medium was supplemented with 2 mM L-glutamine, Earle’s buffered saline solution adjusted to include 1.5 g/L sodium bicarbonate, 0.1 mM non-essential amino acids, 1.0 mM sodium pyruvate, 10% fetal bovine serum, and a 1% mixture of penicillin–streptomycin antibiotics. For experimental use, cells were collected, concentrated through centrifugation, and then re-suspended in sterile saline. Before injection, they were combined with Matrigel® to achieve a final concentration of 50%.

Female athymic nude mice, around 6 weeks old, were given a period of three to seven days for acclimatization prior to the subcutaneous injection of U-87 MG cells into each flank. For the procedure, the mice were anesthetized using inhaled isoflurane. Each flank received an injected with 2 million U-87 MG cells in 50% Matrigel. Prior to treatment initiation, the mice were grouped based on tumor size. Each treatment group, consisting of 5 mice, was collectively housed in a plastic cage positioned at the center of the mouse coil. Tumor measurements were recorded using calipers, and a tumor volume was calculated using the formula 1/2 (a x b2), where b is the smaller of two perpendicular diameters. Using a t-test, the mean tumor volumes between groups were compared. At the end of tumor growth experiments, tumors were removed surgically and weighed immediately. The average tumor weights across groups were compared utilizing the non-parametric Mann Whitney test. Descriptive statistics were then generated for both tumor volume and weight.

### Syngeneic model

Murine experiments were conducted at the UCSD Animal Vivarium, housed within the Moores Cancer Research Center (protocol #141103). All procedures were carried out in compliance with UCSD policies and were approved by the local IACUC. The mice had unrestricted access to food and water and were monitored daily for clinical observations.

GL261 cells were obtained from ATCC (HTB-14, ATCC, Manassas, VA). These cells were cultured in a CO_2_ incubator using Eagle’s Minimum Essential Medium supplemented with 2 mM L-glutamine, Earle’s buffered saline solution adjusted to contain 1.5 g/L sodium bicarbonate, 0.1 mM non-essential amino acids, 1.0 mM sodium pyruvate, 10% fetal bovine serum, and 1% pen-strep antibiotics. For experimental use, the Cells were harvested, centrifuged for concentration, and then resuspended in sterile saline. This suspension was combined with Matrigel to achieve a 50% final concentration.

Before experimentation, female C57/BL6 mice were acclimated for 3 to 7 days. Subsequently, the mice were anesthetized with inhaled isoflurane and received a subcutaneous injection of glioma cells into both flanks – each injection comprised 2 million GL261 cells mixed with 50% Matrigel. Mice were randomized by tumor size prior to initiating treatment. Within each treatment, five mice were co-housed in a centralized plastic cage within the mouse coil. Tumor measurements were recorded using calipers, and a tumor volume was calculated using the formula 1/2 (a x b2), where b represents the shorter of the two perpendicular diameters. The average tumor volumes across groups were statistically compared using a two-sided t-test tests, accounting for unequal variances.

### Generation of ulRFE signals

The method for the generation of signals used in the study are described in a previously published article (Butters et al. 2014). Briefly, EMTx’s molecular interrogation and data system (M.I.D.S.) was used to record the electromagnetic frequencies (EMF) emitted from pharmacological compounds dissolved in a solvent (water, dimethyl sulfoxide [DMSO] or ethanol). These signals were recorded in the time domain and stored in a digital format (WAV) for analysis, selection and testing. The Nyquist rate specifies a sampling rate equal to twice the highest frequency of a given function or signal. With an equal or higher sampling rate, the resulting discrete-time sequence is free of aliasing. All signals were recorded at a 44.1 kHz Nyquist sampling rate (DC – 22.05 kHz).

Mouse sequences from the National Institutes of Health (NIH) Database were used to construct the siRNA for EMulate Therapeutics ulRFE signal measurements and recordings. All siRNA constructs were produced by Dharmacon, a GE Healthcare company.

The compounds that were recorded are as follow:

A. A suspension of paclitaxel (6 mg/ml) in Cremphor EL (the A1A signal).
B. A suspension of CTLA_4 and PD1 siRNAs at 40 uM:
  - Mouse CTLA4 (NM_009843):
    Sense: 5'-GCAGCAUAAGGAUAUAGCAUUAUGG
    Antisense: 3'-CCAUAAUGCUAUAUCCUUAUGCUGCUU
  - Mouse PD1 (NM_008798):
    Sense: 5'-GGAGCAAAUGCCACCUUCACCUGCA
    Antisense: 3'-UGCAGGUGAAGGUGGCAUUUGCUCCCU

## Results

### Xenograft model

**Fig. 2:**
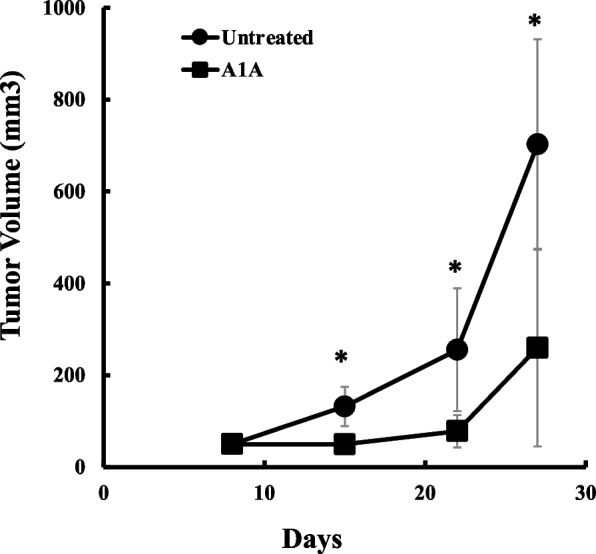
Tumor Volume – U87 Xenograft Study. The mean tumor volume from the xenograft model in A1A-treated mice vs untreated controls. Five animals/treatment group, with tumors in both flanks (*n* = 10 tumors/treatment group). The mean tumor volume of the A1A-treated group was significantly less than that of the untreated control group (mean ± standard deviation on Day 28; untreated: 700 ± 230mm^3^; A1A: 260 ± 220 mm.^3^; *p < 0.001)

**Fig. 3:**
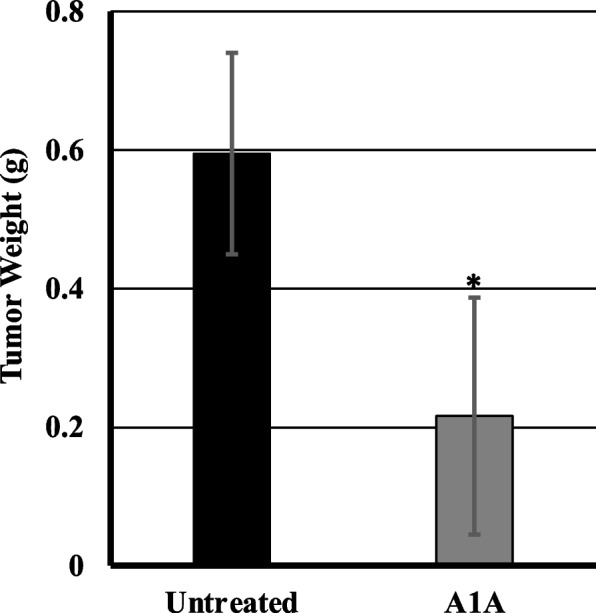
Tumor Weight – U87 Xenograft Study. Mean tumor weight of the excised tumors in the A1A-treated group were significantly less than that of the untreated control group (mean ± standard deviation on Day 28; untreated: 0.6 ± 0.1 g; A1A: 0.2 ± 0.2 g; *p < 0.001)

In the Xenograft model study, we explored the effects of A1A treatment in comparison to untreated controls. Treatment was initiated on Day 8 after the mice were injected with U-87 MG cells. Prior to the introduction of treatment, both groups displayed comparable tumor sizes, with an average volume of 50 ± 13 mm3 for untreated mice and 50 ± 5 mm3 for the A1A-treated mice, as visualized in Fig. 2. Throughout the study, there were no significant clinical observations or mortality among the mice. Interestingly, one week into the treatment, the A1A-treated group exhibited a notable reduction in tumor size compared to their untreated counterparts. This disparity persisted until the study’s conclusion on Day 28, with the untreated group showing a tumor volume of 700 ± 230 mm3 and the A1A-treated group at 260 ± 220 mm3. Further, during necropsy, tumors from the A1A-treated group weighed significantly less, averaging at 0.2 ± 0.2 g, in contrast to the 0.6 ± 0.2 g in the untreated group, as represented in Fig. 3.

### Syngeneic model

**Fig. 4:**
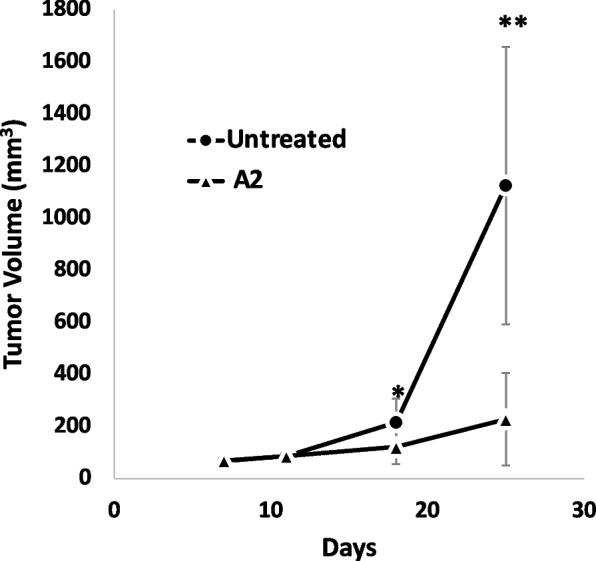
Tumor Volume – Syngeneic Study with GL261. Mean tumor volume from flank syngeneic model. A2-treated mice vs untreated controls. Five animals/treatment group, with tumors in both flanks (*n* = 10 tumors/treatment group). Tumor volume was significantly different between the two treatment groups (mean ± standard deviation on Day 29; untreated: 1750 ± 720 mm^3^; A2: 340 ± 260 mm.^3^; *p < 0.05, **p < 0.001)

In the subsequent Syngeneic model study, we compared the outcomes between mice treated with A2 and untreated controls. Treatment began on Day 7 after injecting the mice with GL261 cells. Pre-treatment data revealed nearly identical average tumor volumes between the two groups, at 67 ± 26 mm^3 for the untreated and 69 ± 22 mm^3 for the A2-treated group, as seen in Fig. 4. As with the previous model, there were no clinically significant changes or fatalities reported throughout the study duration. After 19 days of treatment, a stark contrast in tumor growth emerged. The A2-treated group displayed substantially smaller tumors, measuring at 340 ± 260 mm^3, compared to the untreated group's 1750 ± 720 mm^3, consistent until the study's end on treatment day 29. The outcomes from both models underscore the potential efficacy of A1A and A2 treatments in mitigating tumor growth, paving the way for deeper exploration.

## Discussion

The primary aim of these studies was to determine if the Voyager has a measurable biological effect when used in xenograft and syngeneic models of GBM. Results from the studies strongly suggest that the Voyager can effectively target glioma cells in in vivo experiments. Although murine models are a cornerstone in therapeutic research, providing an initial insight into the effectiveness of new treatment approaches, they do have their limitations.

The flank xenograft models, characterized by the implantation of commercially available immortalized human glioma cells into immuno-deficient mice, are well recognized as a primary screening tool. Their popularity stems from the ease of accessibility of these cells and the models’ inherent simplicity, making it a highly replicable method (Figueroa et al. 2022). However, there is an underlying limitation: they may not always present a reliable predictive outcome and for our 2 signals we have to use different models to show effects. The reason for such unpredictability lies in the potential divergence between the cellular and microenvironmental attributes of these models and actual GBM (Figueroa et al. 2022; Huszthy et al. 2012; Li et al. 2008). Contrarily, patient-derived xenograft models, which utilize tumor cells directly procured during biopsy, are perceived with higher clinical relevance. Their cells tend to echo the defining characteristics intrinsic to human glioma cells. Orthotopic xenograft models, especially those derived from patients, have garnered increased attention in recent times for brain cancer research. Yet, they come with their own set of challenges, mainly due to the cultivation complexities associated with patient-derived cells. This makes them less straightforward and consistent compared to models involving immortalized cells (Figueroa et al. 2022; Taillandier et al. 2003).

On the other hand, syngeneic models, distinguished by the implantation of murine glioma cells into immunocompetent mice, are the gold standard when examining immunotherapy solution (Figueroa et al. 2022). Notwithstanding their utility, drawing a parallel between the responses seen in mouse tumors and those in human clinical settings remains an area of debate.

Adding another layer to the discussion, genetically engineered tumor models, which predominantly house cells exhibiting specific genetic alterations, often fall short in encapsulating the multifaceted heterogeneity of GBM. In comparison to other GBM models, they generally display diminished reproducibility (Figueroa et al. 2022).

In essence, animal models serve as crucial model, offering invaluable insights both in vitro and in vivo, setting the stage for potential therapeutic advancements to progress into human clinical trials. While negative results from these models often signify a potential therapeutic agent's inefficacy in humans, positive outcomes don't unequivocally guarantee success in human trials. However, a favorable result in an animal model does lay a compelling foundation, suggesting that delving deeper into human clinical trials might indeed be a logical subsequent step.

## Conclusion

The results gleaned from our research provide compelling evidence regarding the efficacy of the Voyager in inhibiting tumor growth using murine models of GBM. This significant finding not only underscores the potential therapeutic benefits of the Voyager but also bolsters the rationale for its application in a clinical context. Future preclinical studies will explore mechanism of action and optimizing ulRFE usage in combination studies. Given these promising outcomes, there's a strong case to be made for advancing the Voyager into clinical trials, aiming to further explore its therapeutic implications and potential benefits for patients diagnosed with GBM. As we strive for better treatments in the realm of GBM management, the Voyager presents a promising avenue that warrants thorough investigation in a clinical setting.

## Data Availability

n/a.
